# Displacement, deprivation and hard work among Syrian refugee children in Lebanon

**DOI:** 10.1136/bmjgh-2018-001122

**Published:** 2019-02-04

**Authors:** Rima R Habib, Micheline Ziadee, Elio Abi Younes, Houda Harastani, Layal Hamdar, Mohammed Jawad, Khalil El Asmar

**Affiliations:** 1 Department of Environmental Health, Faculty of Health Sciences, American University of Beirut, Beirut, Lebanon; 2 Public Health Policy Evaluation Unit, Imperial College London, London, UK; 3 Department of Epidemiology and Population Health, Faculty of Health Sciences, American University of Beirut, Beirut, Lebanon

**Keywords:** child labour, occupational health, refugees, children and adolescents, Lebanon

## Abstract

**Background:**

The protracted Syrian war resulted in the largest refugee crisis of our time. The most vulnerable are children who face separation from parents, interruption of schooling and child labour. This study explores the living and working conditions of Syrian children in Lebanon.

**Methods:**

In this cross-sectional study, we randomly selected 153 informal tented settlements and conducted interviewer-administered surveys among Syrian refugee working children in the Bekaa Valley in Lebanon. Those aged 8–18 completed a questionnaire on sociodemographic and occupational characteristics; those aged 4–8 years were surveyed through a household questionnaire.

**Results:**

We surveyed 1902 households, including 12 708 individuals and 4377 working children. Female-headed households were poorer and more food-insecure than male-headed households. Among working children (4–18 years), the average age of starting work was 10.9 years and 74.8% worked in agriculture. Compared with boys, girls earned less and were less likely to be enrolled in school. For 96.3% of working children aged 8–18 years, forced exodus to Lebanon was associated with a first child labour experience. Working conditions were harsh and worse for girls who compared to boys were less likely to receive their salary on time and take time off work. Girls worked longer in the sun and cold and were more likely to report having a health symptom at work, working under pressure and using sharp or heavy objects at work. Seventy-nine children reported knowing another child who died following a work accident.

**Conclusion:**

Children, as young as 4, are forced to work, and many are compelled to forgo educational opportunities in favour of harsh and harmful labour due to difficult economic conditions. State policies facilitating access to work for adult refugees will help families meet basic needs and decrease their dependence on child labour as a coping strategy.

**Key questions**

**What is already known?**

- The war in Syria has forcibly displaced over one million people to Lebanon, including many vulnerable children.
- Research studies on child labour and health are often small, unrepresentative and of low methodological quality.

**What are the new findings?**

- The main finding of this large child labour survey was the disproportionate burden borne by female household heads and working girls.

**What do the new findings imply?**

- Bold interventions are needed to improve the economic opportunities of Syrian refugee households and mitigate their need for working children in Lebanon.
- In addition to such interventions, new labour law provisions that raise the legal age for work and compulsory schooling must be adopted.

## Introduction

The war in Syria has resulted in a crisis of displacement and suffering of catastrophic proportions. Over 11 million Syrians have left their homes since 2011, and the United Nations High Commissioner for Refugees (UNHCR) has registered 5.6 million refugees, about half of whom are under 18 years of age.^1^ The toll of war has claimed many young Syrian lives; those who survived are often exposed to violence, trauma and exploitation.

Syrian refugees constitute a quarter of Lebanon’s population, reaching an estimated 1.5 million, the highest number of refugees per capita in the world.^2^ Over a third of Syrian refugees live in the Bekaa region in eastern Lebanon, a fertile valley home to 42% of the country’s cultivated land that shares a long border with Syria and is characterised by a plethora of informal tented settlements (ITS).^1^ Before the war in Syria, Bekaa had long been a place where Syrian migrants participated in seasonal agricultural work.^3^

Restrictive policies adopted by Lebanon in response to the large influx of Syrian refugees are underpinned by the fact that it is not a signatory to the 1951 Geneva Convention and its 1967 Protocol.^4^ In January 2015, the Government of Lebanon asked that the UNHCR suspend registration of Syrians entering Lebanon, and imposed stricter regulations governing their entry and stay,^4^ including a pledge not to seek work and a $200 yearly residence permit fee.^5^ A 2017 report assessing the vulnerability of Syrian refugees in Lebanon showed that 75% of Syrian refugee households in Lebanon have no access to basic food and shelter, and 58% are living in extreme poverty unable to access the basic needs for survival.^6^ Such poverty cultivates conditions where children must work to ensure their families’ survival.^7^ In the city, children do various street jobs, including begging for money, selling food and drinks, and shining shoes, whereas Syrian working children living in the rural Bekaa are typically employed in the agricultural sector.^8^

Prior to 2011 child labour rate in Syria was 4% (5% male, 3% female) for children aged 5–14 years.^9^ The Syrian war has been associated with a rise in child labour both inside and outside Syria.^10 11^ More recently, a 2011 study of 192 working children in Bekaa found that 140 were Syrian and 36.5% were under 13 years of age.^12^ The full extent of child labour among Syrian refugees in the Bekaa, however, has not been studied, but it is likely widespread.

Studies of child labour and health are generally limited to small, unrepresentative samples of low methodological quality that enquire about work-related physical injury and harmful exposures, nutritional health, and psychosocial health.^13 14^ The Middle East is one of several understudied regions with respect to child labour, which is disconcerting given that regional instability is likely to encourage child labour. The objective of this study is, therefore, to describe the housing, sociodemographic and occupational characteristics of working Syrian refugee children in the Bekaa Valley of Lebanon, and provide the first comprehensive account of their situation.

## Methods

The study was approved by the Institutional Review Board (IRB) at the American University of Beirut (IRB Protocol Number: FHS.RH1.08) and follows the Strengthening the Reporting of Observational Studies in Epidemiology guidelines (online supplementary appendix).

10.1136/bmjgh-2018-001122.supp1 Supplementary data

### Design, setting and sampling

This study was a cross-sectional household survey of Syrian refugee working children residing in ITS in Lebanon. We used the Interagency Mapping Platform (IAMP),^15^ a database of 234 546 Syrian refugees in Lebanon living in 6192 ITS, to develop the sample frame. The IAMP is used to coordinate humanitarian activities in Lebanon; it contains information on all ITS and their residents in the country, regardless of their documentation status.^15^ Non-governmental agencies conduct the mapping of ITS across Lebanon under the oversight of Medair, a humanitarian organisation operating in the country.^16^ The IAMP database is updated on a 3-month basis.^16^ We used the latest accessible version at the time of sampling. Our frame included four districts (Baalbek, Hermel, West Bekaa and Zahle) from the Bekaa and Baalbek-Hermel governorates, areas home to 183 816 Syrian refugees living across 3748 ITS. We calculated a sample size of 1884 households based on a 17% estimated prevalence of child labour.

We randomly selected 153 ITS and generated tent lists through discussion with a local job finder and community gatekeeper (the ‘shaweesh’) to identify households containing all working children aged 4–18. The shaweesh is a member of the Syrian refugee population who acts as a middleman in each ITS, renting tents to refugees and hiring children to work in nearby farms, restaurants, auto repair shops or other workplaces.^17^ Shaweeshs are also well-connected community leaders; they often act as mediators between aid organisations and refugee households.^18^ During the fieldwork, when the shaweesh was not sure which tents housed working children, all the tents in the ITS were approached by the study team to identify and enumerate those where working children reside. Each eligible tent was visited at most three times before being considered unavailable. We recruited and trained 33 male and female fieldworkers (Lebanese and Syrians) of whom 27 were selected for fieldwork. A number of fielworkers had previous experience in survey administration, especially with Syrian refugees in the Bekaa Valley. Fieldworkers attended a 7-day training workshop before visiting ITS in 2017. The training focused on the objectives and purpose of the study, interviewing techniques, filling electronic questionnaires, roles and duties of data collectors, administering informed consent, and handling and reporting child abuse. Data collectors sought oral informed consent from the female homemaker and assent from the working children aged >8 to ≤18. They explained the purpose of the study, that all data will be kept confidential and anonymous, and that refusal to participate would not adversely affect their relationship with humanitarian, academic or governmental institutions involved in the study. No financial compensation was given to study participants.

### Questionnaire and measures

We developed a child and household questionnaire, both face-to-face and interviewer-administered using electronic tablets, which we prepiloted and translated into colloquial Arabic. The child questionnaire was for child workers aged >8 to ≤18 and gathered sociodemographic, physical and mental health,^19^ and detailed work history indicators. The household questionnaire asked both individual-level questions, including a shorter version of the child questionnaire for child workers aged ≥4 to ≤8, and household-level questions. The female homemaker was targeted for completion of the household questionnaire based on previous research experience from surveys in Lebanon where she was considered more knowledgeable of household characteristics and household members’ issues.^20–23^ If the female homemaker was not available, we interviewed an adult household member.

The respondent to the household questionnaire answered questions on behalf of child workers aged ≥4 to ≤8 years and on behalf of other individuals residing in the same dwelling for at least 15 days prior to the survey, including sociodemographic and health indicators. Data collected on households included household expenditure and indices on housing and infrastructure problems, assets and food security (Reduced Coping Strategy Index [RCSI] and Livelihood Coping Strategy Index [LCSI]).^24 25^ Detailed descriptions of these measures and quality control processes are outlined in the online supplementary appendix.

### Statistical analysis

We performed a descriptive analysis, reporting frequencies and percentages for categorical data and means and SD for continuous data. We reported household characteristics by the sex of household head and their working status, and reported sex-stratified sociodemographic and occupational characteristics of the working children. Differences were tested for using either χ^2^ or independent-samples t-tests. We constructed a linear regression model to test the association between the wage of working children and sex, adjusting for field of work. We considered an alpha value of 0.05 as statistically significant and conducted all analyses on Stata V.15.0. Missing data were minimal, and observations with missing data were not dropped from the analysis. We used the programming language R (V.3.3.2) to generate graphical illustrations of the data.

## Results

### Total survey population

The total survey population consisted of 12 708 individuals; these household members were 47.5% male, 65.2% aged 18 years or under, and 69.9% single. The vast majority (83.6%) could not read or write, or had attained elementary school education, and 54.9% of adults were unemployed.

### Household characteristics

We surveyed 1902 households, nearly all (97.7%) of which were makeshift tents. The average number of residents and working children per household was 6.7 and 2.4, respectively. The monthly household income and expenditure per capita was US$50.7 and US$119.7, respectively, indicating a monthly income-expenditure gap of US$69.0. About two in five households had a high number of housing and infrastructure problems. A majority (74.3%) of households were severely food-insecure. Furthermore, 76.6% had high levels of reduced coping (RCSI ≥10), and 38.1% adopted emergency coping strategies (LCSI 4).

**Figure 1:**
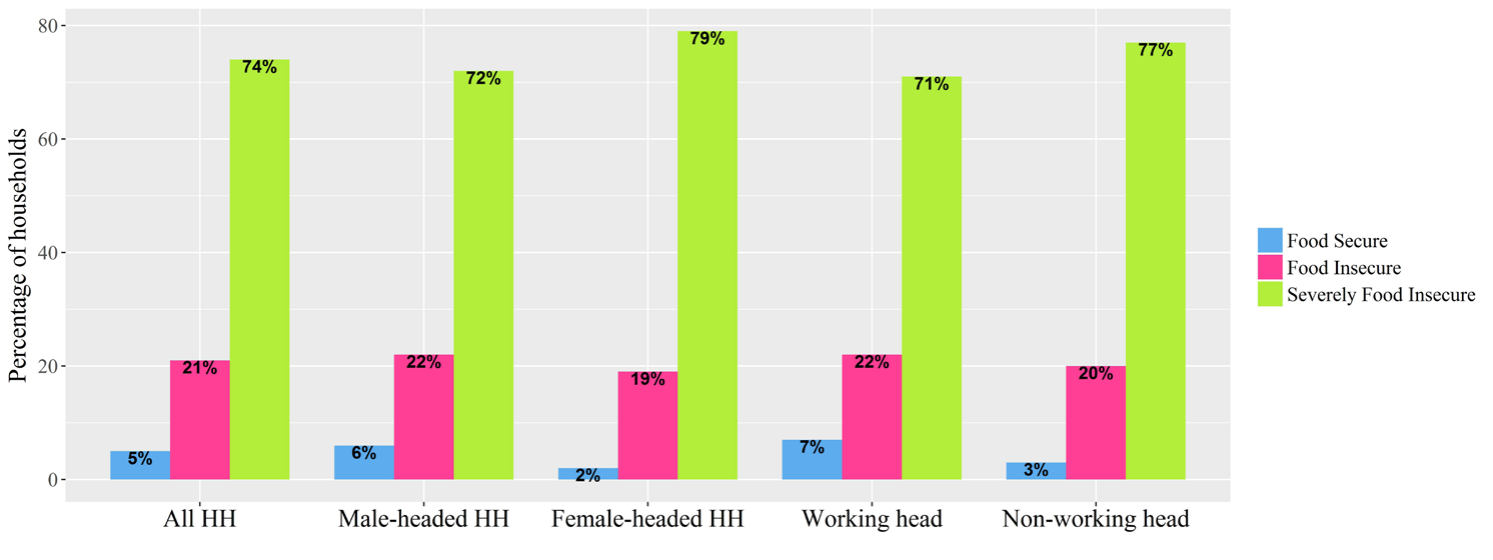
Food security by sex and working status of houshehold head (HH).

These characteristics are stratified by the household head’s sex and working status in table 1. About 70.9% of households were headed by men, and 54.6% of household heads did not work. A disproportionate burden on female-headed households was apparent: compared with male-headed households, they had a larger income-expenditure gap (US$86.0 vs US$62.2) and greater food insecurity. Furthermore, being in the lowest assets index was twice as common in female-headed households as in male-headed households (34.7% vs 19.5%). Similarly, the differences in households with working versus non-working heads were stark: compared with households with working heads, those with non-working heads had half the household income per capita but similar household expenditure per capita, and greater food insecurity (figure 1).

**Table 1:** Household characteristics stratified by sex and working status of the household head (n=1902)

	Full sample		Male-headed		Female-headed		P value	Working head		Non-working head		P value
Total, n	1902		1349		553			863		1039		
Socioeconomic characteristics	**Mean**	**SD**	**Mean**	**SD**	**Mean**	**SD**		**Mean**	**SD**	**Mean**	**SD**	
Age of household head, years	42.8	10.3	43.2	10.4	41.8	10.2	<0.001	38.8	9.3	46.1	10.0	<0.001
Household size	6.7	2.6	7.1	2.6	5.7	2.4	<0.001	6.4	2.5	6.9	2.7	<0.001
Number of children	3.9	1.9	4.0	2.0	3.6	1.9	<0.001	3.8	1.9	3.9	2.0	0.2065
Number of working children	2.4	1.3	2.4	1.3	2.5	1.3	0.3047	2.4	1.3	2.5	1.3	0.1484
Monthly household income per capita, US$	50.7	43.6	48.3	44.1	56.3	41.9	<0.001	68.5	50.0	35.9	30.4	<0.001
Monthly household expenditure per capita, US$	119.7	210.6	110.5	86.5	142.3	365.8	<0.01	122.8	186.6	117.1	228.8	0.558
	**N**	**%**	**n**	**%**	**n**	**%**		**n**	**%**	**n**	**%**	
Housing problems index												
Low levels of housing problems (<7)	329	17.3	230	17.0	99	17.9	0.903	174	20.2	155	14.9	<0.01
Medium levels of housing problems (7–8)	754	39.6	537	39.8	217	39.2		347	40.2	407	39.2	
High levels of housing problems (>8)	819	43.1	582	43.1	237	42.9		342	39.6	477	45.9	
Infrastructure problems index												
Low levels of infrastructure problems (<4)	201	10.6	154	11.4	47	8.5	<0.01	90	10.4	111	10.7	0.905
Medium levels of infrastructure problems (4–6)	764	40.2	571	42.3	193	34.9		343	39.7	421	40.5	
High levels of infrastructure problems (>6)	937	49.3	624	46.3	313	56.6		430	49.8	507	48.8	
Assets index												
First quartile (poorest)	455	23.9	263	19.5	192	34.7	<0.001	240	27.8	215	20.7	<0.001
Second quartile	467	24.6	320	23.7	147	26.6		179	20.7	288	27.7	
Third quartile	483	25.4	350	25.9	133	24.1		215	24.9	268	25.8	
Fourth quartile (richest)	497	26.1	416	30.8	81	14.6		229	26.5	268	25.8	
Reduced Coping Strategy Index (RCSI)												
No or low reduced coping (RCSI 0–3)	157	8.3	116	8.6	41	7.4	0.183	94	10.9	63	6.1	<0.001
Medium reduced coping (RCSI 4–9)	288	15.1	215	15.9	73	13.2		140	16.2	148	14.2	
High reduced coping (RCSI ≥10)	1457	76.6	1018	75.5	439	79.4		629	72.9	828	79.7	
Livelihood Coping Strategy Index (LCSI)												
Household not adopting coping strategies (LCSI 1)	24	1.3	22	1.6	2	0.4	0.062	18	2.1	6	0.6	<0.01
Stress coping strategies (LCSI 2)	87	4.6	57	4.2	30	5.4		50	5.8	37	3.6	
Crisis coping strategies (LCSI 3)	1066	56.0	746	55.3	320	57.9		466	54.0	600	57.7	
Emergency coping strategies (LCSI 4)	725	38.1	524	38.8	201	36.3		329	38.1	396	38.1	

### Working children aged ≥4 to ≤18 years

We surveyed 4377 working children, 52.2% of whom were male, with a mean age of 12.8 (SD=3.1) years, and 96.6% were single. Illness or disability was reported among 18.5% of the working children. Table 2 presents more detailed occupational and educational indicators. The average age for starting work in Lebanon was 10.9 years. Girls were older and started work later than boys by 0.4 years. Girls also worked for a slightly longer duration (1.5 vs 1.4 years) and worked less hours per day (6.4 vs 6.7 hours). They appeared to have a higher monthly wage than boys (US$74.9 vs US$69.7), but when controlling for age, field of work, length of stay in the neighbourhood and geographical location in a regression model, girls actually earned less than boys (beta coefficient −0.06, 95% CI −0.09 to 0.03, p<0.001). Girls were also more likely to give their wages to their parents (61.7% vs 54.3%), not take any time off work (62.0% vs 55.2%) and do more household chores per week than boys (9.9 vs 3.0 hours).

**Table 2:** Distribution of occupational and educational outcomes of working children >4 to ≤18 years stratified by sex (n=4377)

	Full sample		Male		Female		P value
Total, n	4377		2285		2092		
	**Mean**	**SD**	**Mean**	**SD**	**Mean**	**SD**	
Age, years	12.8	3.1	12.6	3.1	13.0	3.0	<0.001
Age started working, years	10.9	2.9	10.7	2.9	11.1	2.8	<0.001
Duration of work, years	1.5	1.4	1.4	1.3	1.5	1.4	<0.05
Hours worked daily	6.6	2.6	6.7	2.7	6.4	2.6	<0.001
Hours doing housework weekly	6.3	7.1	3.0	4.3	9.9	7.8	<0.001
Past 30 days wage, US$	72.2	58.9	69.7	61.8	74.9	55.5	<0.01
	**N**	**%**	**n**	**%**	**n**	**%**	
Suffering from illness or disability	808	18.5	424	18.6	384	18.4	0.865
Currently enrolled in school	800	18.3	447	19.6	353	16.9	<0.05
Ever injured at work	1326	30.3	785	34.4	541	25.9	<0.001
Uses sharp or heavy objects at work	1483	33.9	731	32.0	752	35.9	<0.01
Gives wages to parent*	2303	57.8	1142	54.3	1161	61.7	<0.001
Takes time off work weekly†	1818	41.5	1024	44.8	794	38.0	<0.001
Reason for not attending school‡							
I started working.	1803	50.5	902	49.2	901	51.8	<0.001
There isn’t a nearby school.	532	14.9	304	16.6	228	13.1	
I don’t have time to go to school.	369	10.3	177	9.6	192	11.0	
My parents were unable to pay.	269	7.5	136	7.4	133	7.7	
I don’t feel it is useful/I don’t like it.	257	7.2	146	8.0	111	6.4	
Other	343	9.7	170	9.3	173	9.9	
Decision to work taken by:							
Person himself/herself	2619	59.8	1404	61.4	1215	58.1	<0.001
Mother	886	20.2	417	18.2	469	22.4	
Father	765	17.5	423	18.5	342	16.3	
Other	107	2.5	41	1.9	66	3.1	
Reason to start work§							
To support the family	3748	85.6	1946	85.2	1802	86.1	0.359
To earn money	2703	61.8	1425	62.4	1278	61.1	0.387
Field of work§							
Agriculture	3275	74.8	1497	65.5	1778	85.0	<0.001
Waste picking	232	5.3	164	7.2	68	3.3	<0.001
Construction	149	3.4	149	6.5	0	0.0	<0.001
Street services	121	2.8	79	3.5	42	2.0	<0.01
Car wash	100	2.3	97	4.3	3	0.1	<0.001
Mechanics	79	1.8	79	3.5	0	0.0	<0.001
Other	494	11.3	287	12.6	207	9.9	<0.01

Due to respondents answering ’I don’t know’ or respondents only answering parts of the survey depending on earlier answers, the total numerators/denominators are *3985/3985, †4376/4377 and ‡3573/3577.

§Total greater than 100% as more than one option possible.

Only 18.3% of working children were enrolled in school, with slightly fewer girls enrolled than boys (16.9% vs 19.6%); the main reason for not enrolling in school was starting work, reported by 50.5% of all children. Those who did go to school often went to public (58.0%) or non-formal (36.8%) schools. The decision to start working was taken either by the child himself/herself (59.8%) or by the parents (47.7%), with the majority (85.6%) reporting family support as the main reason for starting work. Nearly three-quarters (74.8%) reported working in agriculture; this was substantially higher among girls than boys (85.0% vs 65.5%). About a third of the sample (30.3%) reported ever being injured at work, which was slightly higher in boys than in girls (34.4% vs 25.9%), and about a third (33.9%) reported using sharp and heavy objects at work (35.9% for girls vs 32.0% for boys).

### Working children aged >8 to ≤18 years

The subsample of working children aged >8 to ≤18 years, totalling 4090, answered a separate and more comprehensive survey. Of these children, 15.6% had been in Lebanon for less than a year, 51.9% for 1–5 years and 32.5% for more than 5 years. About a third (33.7%) lived without their father, in 40.4% of cases this was because he was either dead or missing; 7.9% lived without their mother, in 42.8% of cases this was because she was still in Syria. Nearly all working children (91.3%) cited the Syrian war as the reason for moving to Lebanon, and 84.5% of those who went to school in Syria no longer went to school in Lebanon. Moreover, for 96.3% of working children, the forced exodus to Lebanon was associated with a first child labour experience.

More detailed health and occupation characteristics of working children aged >8 to ≤18 years are presented in table 3. The results show the degree of harshness in their working conditions. A majority (82.4%) reported working in the sun for an average of 5.9 hours per day; both the prevalence and intensity were significantly higher among girls. Furthermore, 29.7% reported working in the cold for an average of 5.8 hours per day and 10.6% reported working under the rain.

**Table 3:** Health and occupational characteristics of working children aged >8 to ≤18 years, stratified by sex (n=4090)

	Total		Male		Female		P value
Total, n			2107		1983		
	**Mean**	**SD**	**Mean**	**SD**	**Mean**	**SD**	
Weather exposure							
Hours/day of work in the sun	5.9	2.2	5.8	2.3	6.1	2.2	<0.01
Hours/day of work in the cold	5.8	2.4	5.7	2.5	5.9	2.3	0.173
Well-being indices (range)							
Child Satisfaction Index (0–9)	3.9	2.6	3.8	2.6	3.9	2.5	0.2363
Child Well-Being Index (0–16)	8.5	3.0	8.4	3.0	8.6	3.0	0.1096
Child Perception Index (0–6)	1.3	1.6	1.2	1.6	1.3	1.7	0.05
Child Optimism Index (0–10)	3.9	2.1	3.8	2.1	3.9	2.1	0.2345
	**n**	**%**	**n**	**%**	**n**	**%**	
Receive salary on time*							
No	1394	37.7	626	32.6	768	43.3	<0.001
Yes	2302	62.3	1297	67.4	1005	56.7	
Ever health symptom at work							
No	192	4.7	117	5.6	75	3.8	<0.01
Yes	3898	95.3	1990	94.4	1908	96.2	
Allowed breaks at work†							
No	751	18.4	403	19.1	348	17.6	0.196
Yes	3334	81.6	1702	80.9	1632	82.4	
Allowed to eat at work‡							
No	1365	33.4	670	31.8	695	35.0	<0.05
Yes	2723	66.6	1435	68.2	1288	65.0	
Water bottles drunk at work§¶							
0–1 bottle per day	1057	26.0	494	23.6	563	28.6	<0.001
2 bottles per day	1227	30.2	600	28.7	627	31.8	
3 or more bottles per day	1781	43.8	999	47.7	782	39.7	
Lifting weights more than 25 kg							
No	2625	64.2	1131	53.7	1494	75.3	<0.001
Yes	1465	35.8	976	46.3	489	24.7	
Work under pressure to finish job on time							
No	1814	44.4	969	46.0	845	42.6	<0.05
Yes	2276	55.6	1138	54.0	1138	57.4	

Due to respondents answering ‘I don’t know’ or respondents only answering parts of the survey depending on earlier answers, the total numerators/denominators are *3696/3699, †4085/4090, ‡4088/4090 and §4089/4090.

¶Each bottle is 0.5 L.

**Figure 2:**
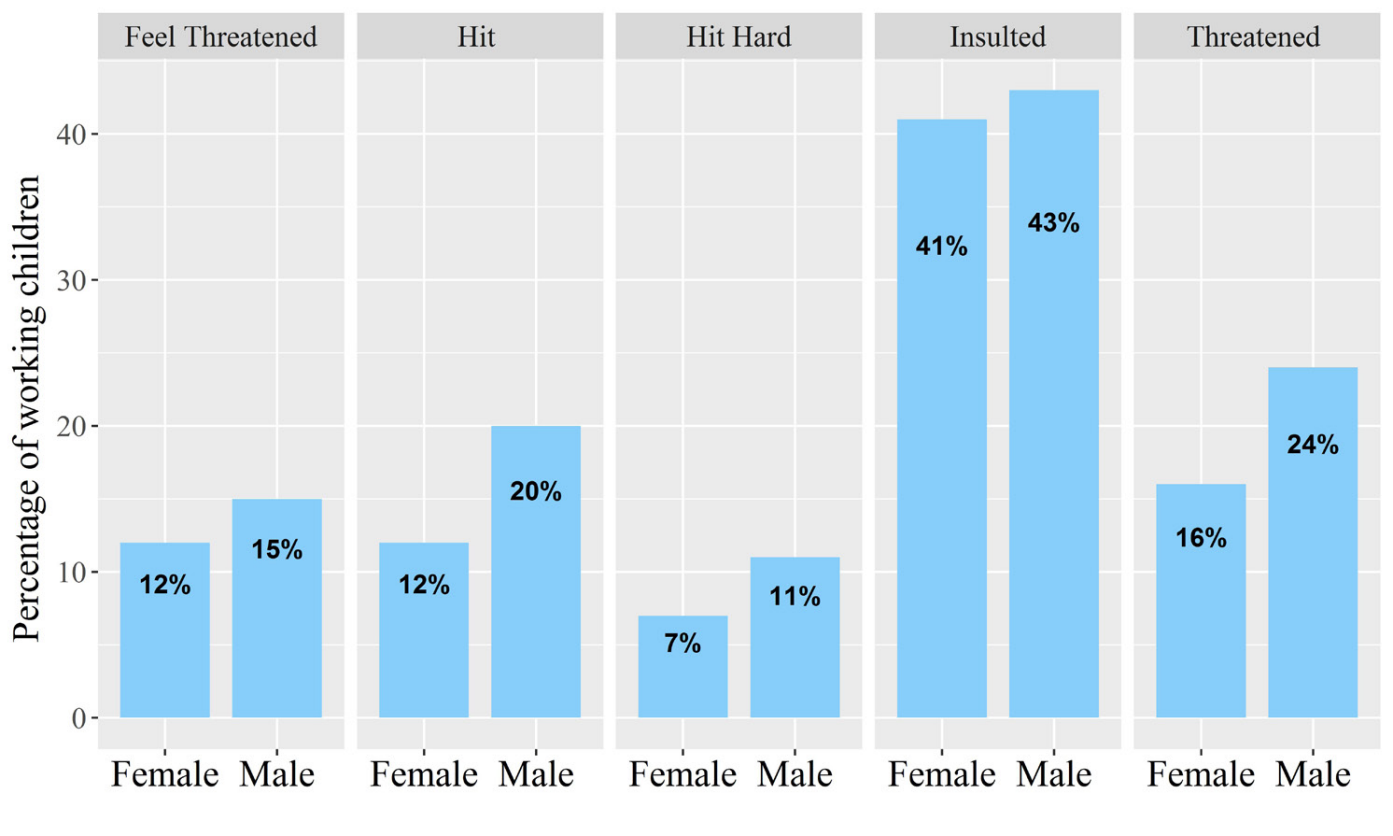
Mistreatment at the workplace among working children aged >8 to ≤18 years, by sex. P values for χ^2^ difference in proportions: feel threatened:<0.05; hit: <0.001; hit hard: <0.001; insulted: NS; threatened: <0.001.

Girls were more likely to be exploited at work than boys. In addition to our findings that they earned a lower salary than boys, girls were also less likely than boys to receive their salary on time (56.7% vs 67.4%) and to drink three or more bottles (0.5 L each) of water per day (39.7% vs 47.7%). Moreover, girls were more likely than boys to report a health symptom at work (96.2% vs 94.4%) and to work under pressure to finish their job on time (57.4% vs 54.0%). The most common health symptoms experienced at work were fatigue (93.1%), fever (53.3%) and body weakness (41.7%). Also, girls reported having more health symptoms than boys (4.9 vs 4.4, p<0.001). Physical and verbal abuse experienced by child workers was apparent; over 40% reported being insulted at least once at work, and some were even hit hard and threatened (figure 2). In total, 45.6% of working children reported at least one form of physical or verbal abuse. Seventy-nine working children reported knowing another child who had died following a work accident.

The financial cost of injury was also apparent. Of working children, 63.9% reported that treatment of work injuries is paid for by parents, 22.2% by the child himself/herself and 13.5% by the employers. Healthcare costs related to workplace injuries were paid for using cash 86.2% of the time, and among these cash payers 86.8% took out loans to cover the expense.

**Figure 3:**
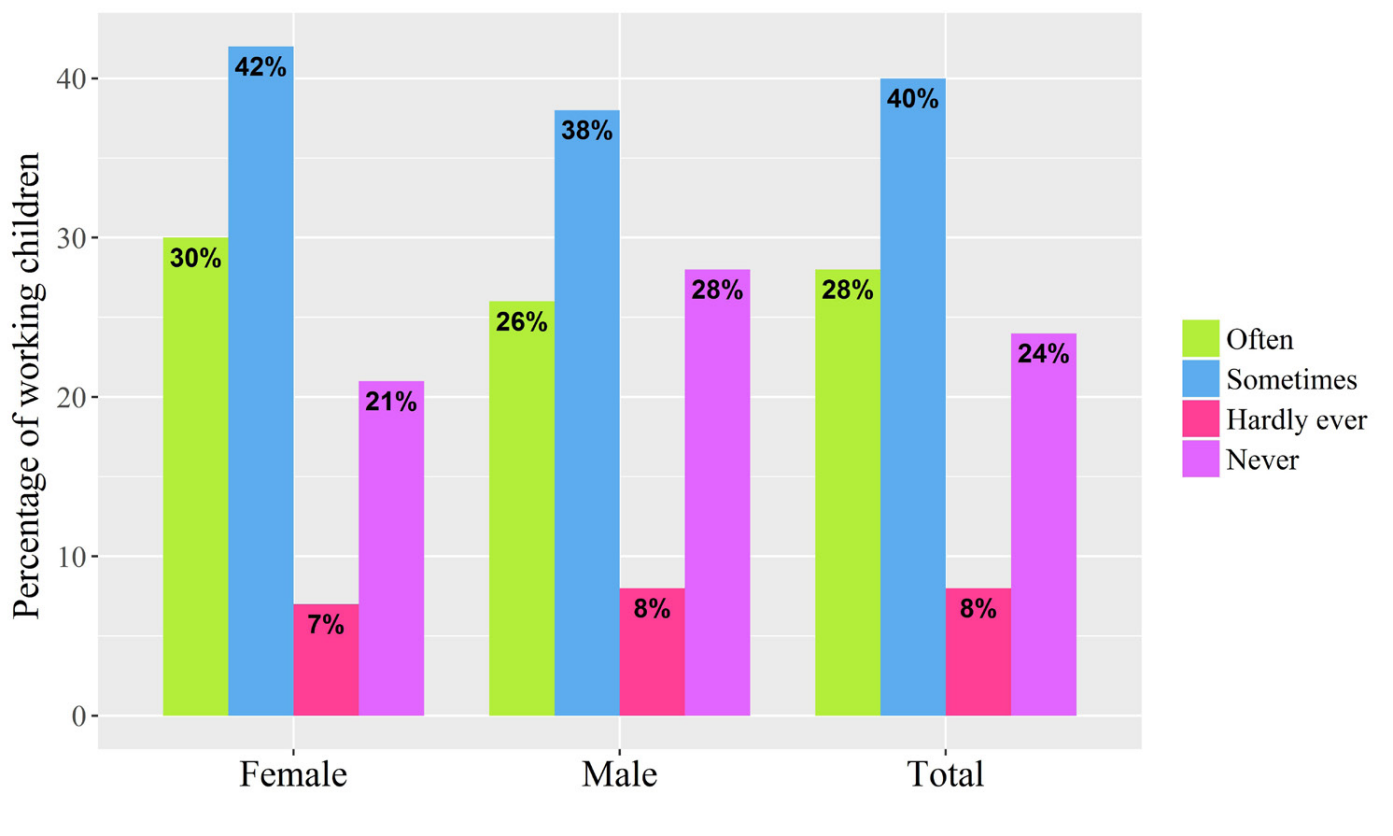
Feelings of loneliness among working children aged >8 to ≤18 years, by sex.

The health and occupational characteristics of working Syrian refugee children in Lebanon are likely to impact mental health. One example is the high proportion (68%) reporting feelings of loneliness sometimes or often (figure 3). Different indices of mental health, including satisfaction, well-being, future perception and optimism, did not differ by sex (table 3).

## Discussion

This study, one of the largest and most comprehensive child labour surveys globally,^13 14^ describes the dire housing, sociodemographic and occupational characteristics of Syrian refugee children in the Bekaa Valley of Lebanon. Children, some as young as 4, are forced to work, and many are compelled to forgo educational opportunities in favour of harsh and potentially harmful labour.

Our main finding was that females, whether household heads or working children, were disproportionately burdened with hardships. Female-headed households were poorer, more food-insecure and had greater expenditures than male-headed households. Working girls earned less money per month than boys once adjusting for age, field of work, length of stay in the neighbourhood and geographical location; they were less likely to be enrolled in school and were more exploited at work. These alarming educational and occupational patterns could negatively affect the future prospects of war-displaced child workers in Lebanon, particularly girls. Lower literacy might lead to less earning potential, which may perpetuate poverty.

Inaction to the plight of child refugee workers in Lebanon has both regional and global implications. Lebanon currently has the highest number of refugees per capita in the world,^5^ and the influx of Syrian refugees places pressure on an already fragile infrastructure and economy.^5 26^ The lack of economic opportunities, reflected by the high rate of unemployment of household members, degree of food insecurity and the need for child labour,^27 28^ is symptomatic of a precarious and arduous way of life for Syrian refugees. The protracted nature of the war in Syria, the diminished opportunity for repatriation and the reduction of international aid may have drained familial assets and cultivated conditions rife for child labour.

Strong political reverberations to the Syrian refugee crisis are also felt further afield. The question of migrants, the facilitation of their movement and their settlement are creating much debate and discord within and between the Member States of the European Union regarding migration policies.^29 30^ The management and support of migrants require dealing with various issues: political, social and economic. The case of working Syrian refugee children in Lebanon is representative of such complexities and challenges.

Child workers and their Syrian refugee households in the Bekaa Valley must be better supported politically and humanitarianly. New child protection policies should be drafted and enforced, and collaboration between the Government of Lebanon and the humanitarian sector should prioritise the protection of this vulnerable population group. Legal frameworks that protect and support Syrian child workers should be met with scalable development projects and economic opportunity for households. Such interventions will improve the social and educational capital among children. Enabling conditions where Syrian refugee families can provide for their children will mitigate the need for child labourers.

Legal frameworks can include temporary and affordable work and residency permits that can widen employment opportunities and recognise Syrian refugees’ status in Lebanon. The current Lebanese labour law^31^ prohibits the work of children under 14 years. The government should adopt the revised law on child labour, particularly the provisions on raising the age of compulsory education and the minimum legal age for work to 15 years.^32^ A review of legislation governing refugee rights that align with values of the Lebanese state is needed. Workplace interventions should train employers on child abuse and occupational health and safety, and wider development interventions should prioritise income-generating projects, food security projects, and free and accessible educational opportunities, including expansion of vocational training. Support packages for families whose children are enrolled in school could encourage enrolment and give families a motivation and practical solutions to choose schooling over child labour.

This study is not without its limitations. Despite employing methodologically robust cross-sectional methods, including sample frames, random sampling and quality control, associations cannot infer causation. Generalisability may be limited to other settings, but given the breadth of questions asked and that this sample is one of the largest among child workers globally,^13 14^ much learning can be taken to other settings in support of understanding the complex hardships faced by refugee child workers. The use of community gatekeepers to identify households with working children is a possible source of bias. However, we tried to compensate for this bias by enumerating all tents in the ITS when the shaweesh was in doubt. Another limitation of this study is that it did not enquire about sexual harassment to which children, and girls in particular, might be exposed. While not a national study, the Bekaa Valley hosts the largest number of Syrian refugees in Lebanon and hence is representative of the conditions of Syrian refugee children in the country.

## Conclusion

The difficult truths revealed by this research show the extent and impact of child labour on Syrian refugees. The harsh and hazardous work conditions constitute a major challenge, as reversing the factors that led to a child worker culture requires a coordinated effort across international, national and local cleavages. Solutions are difficult but not impossible. A number of interventions could be adopted, including creating employment opportunities for adults, targeting food-insecure families with food aid programmes, organising child safety trainings and awareness campaigns, and encouraging schooling opportunities through motivational assistance packages for families with enrolled children. More research is needed to inform policies and intervention strategies that account for the complexities of protracted refugee situations. Furthermore, to address the challenges imposed by mass migration on a global scale, problems must be resolved in host countries where migrants are facing difficulties to survive.
