# Supplementary material for: Displacement, deprivation and hard work among Syrian refugee children in Lebanon
Source: BMJ Glob Health. 2019 Feb 4;4(1):e001122. doi: 10.1136/bmjgh-2018-001122 (PMC6528770; doi:10.1136/bmjgh-2018-001122)
Supplement: Supplementary data [file bmjgh-2018-001122supp001.pdf]

ADDITIONAL METHODOLOGICAL DETAILS

Housing problems

A housing problems index was developed using the following 14 items:

- Lack of sunlight entering the house
- Lack of wind entering when windows are open
- Humidity
- Holes or cracks in the walls
- Holes or cracks in the ceiling
- Leakage from the walls
- Uncovered electric wires
- Ants/cockroaches or any other insects
- Rats/mice
- Venomous insects or animals (scorpions, snakes…)
- Heating the home using fuels other than gas/electricity/solar power
- Heating water using fuels other than gas/electricity/solar power
- Cooking using fuels other than gas/electricity/solar power
- Shared or no bathroom

The presence of each item was given a score of one, so the maximum score was 14. We categorised the housing problems index into six problems or less, seven to eight problems, and more than eight problems.[1-3]

Infrastructure problems

An infrastructure problems index was developed using the following 17 items:

- Flood in the household
- Forced to evacuate because of flood
- No electricity supplied to the household
- Less than 4 hours of electricity per day
- No heating supply during winter
- Complaint of service water scarcity
- Dissatisfaction with the quality of service water used
- Complaint of drinking water scarcity
- Dissatisfaction with the quality of water received
- Does not make water safe for drinking
- Water not reaching the kitchen directly
- Water not reaching the toilet directly
- Water not reaching the shower directly
- Disposing household garbage randomly or in a nearby river
- Disposing used service water in the street or nearby river
- Disposing wastewater in the street, a nearby river, or open bins
- Bad smells

The presence of each item was given a score of one, so the maximum score was 17. We categorised the infrastructure problems index into three problems or less, four to six problems, and more than six problems.[1-3]

Assets

A household assets index was calculated based on several questions about ownership of 27 items (goods and commodities) such as: bed, washing sink, cooking stove, refrigerator, heater, television, radio, car, telephone line, and others. A principal components analysis was conducted where indicators of household assets were converted into z-scores and factor loadings for a single assets factor were calculated. For each respondent, values of the indicator variables were multiplied by the factor loadings to obtain an assets score. Respondents were grouped into quartiles by assets score, with the lowest quartile considered the poorest.

Food security

Food security was firstly measured through the Reduced Coping Strategies Index (RCSI), which includes five individual coping behaviours (eating less preferred foods, borrowing food/money from friends and relatives, limiting portions at mealtime, limiting adult food intake, and reducing the number of meals per day). The RCSI is used to measure the presence and severity of food security across different contexts and settings.[4]

Food security was also measured through an adapted version of the Livelihood Coping Strategy Index[5] using ten long-term coping strategies:

- Selling household goods
- Spending some or all household savings
- Buying food on credit or borrowing money to purchase food
- Borrowing money
- Selling productive assets or means of transport
- Reducing essential non-food expenditures
- Withdrawing children from school
- Selling house or land
- Asking for money from strangers
- Accepting high risk or exploitative temporary jobs

Health symptoms at work

Health symptoms experienced at work were listed in the survey including: fatigue, fever, body weakness, imbalance, tachycardia, cramps, physical breakdown, quivers/chills, chest pain, yellow/pale skin, dehydration, cold sweat, difficulty in concentration/memory loss, nose bleed, skin allergies, respiratory problems, stomach problems, urinary problems, and others. Each symptom was transformed into a binary variable (No: 0, Yes: 1). The sum of all 19 symptoms was computed for each child, then the mean number of symptoms was computed by sex.

Work injuries

Working children responding “Yes” to any of the following injuries were considered to have a work injury.

- Fell off a ladder
- Bit by a snake
- Bit by insects
- Bit or kicked by animals
- Severely wounded
- Heavy object fell on head/body
- Electrocuted
- Fell off a tree
- Fell from heights
- Nail/shard punctured foot/body
- Burnt
- Fracture/sprain
- Eye problems
- Others

Child satisfaction

A Child Satisfaction Index was developed from twelve statements that asked about life satisfaction across various areas:

- The quality of your education at school?
- Your relationship to your teachers?
- The financial situation of your family?
- Your health?
- Your weight?
- Your life?
- Your school?
- Future study opportunities?
- Future work opportunities?
- Leisure time opportunities?
- Opportunities to take part in social/ activities?
- Opportunities to voice your opinion in your school or community about issues that affect young people?

Responses were structured as a four point Likert scale (very satisfied, satisfied, not so satisfied, and not satisfied at all). Respondents answering “Not so satisfied” or “Not satisfied at all” were given a score of one; the remainder were given a score of zero. A higher score was indicative of having less satisfaction with life.

Child wellbeing

A Child Wellbeing Index was developed from 16 statements that asked about wellbeing:

- I feel alone these days

- No matter how hard people try in life, it does not make any difference
- I feel discriminated against
- My whole world feels like it is falling apart
- I wish I were someone important
- It is hard for me to tell what is right and wrong these days
- I don't like to live by society's rules
- These days there are no clear moral standards and norms
- I often wonder what the meaning of life really is
- Most people today know where they stand and where they are going to
- I am optimistic about my future
- The majority of youngsters are lost and do not know what they want
- Nowadays only money is important here
- In Lebanon, opportunity is not always available for those who try to strive ahead
- Young men and women in my community have a reasonable good chance of reaching their goals in life
- Nowadays only dishonest people are successful

Responses were structured as a four point Likert scale (strongly disagree, disagree, agree, strongly agree). Respondents answering "Agree" or "Strongly Agree" were given a score of one; the remainder were given a score of zero. A higher score was indicative of worse child wellbeing.

Child perception

A Child Perception Index was developed from six statements that asked about perceptions of the future:

- I will learn the craft I like
- I will receive the degree I like
- I will find a job I have a degree for
- I will become a successful person
- I will remain jobless for a long time
- My future will become better

Responses were structured as either "Agree", "Disagree", or "I don't know". There were minimal respondents who answered "I don't know", so respondents who answered negatively about their perception of the future were given a score of one for each statement. A higher score was indicative of a more negative perception of the future.

Child optimism

A Child Optimism Index was development from 10 statements adapted from the Life Orientation Test-Revised.[6]

- In uncertain times, I usually expect the best
- It's easy for me to relax
- If something can go wrong for me, it will
- I'm always optimistic about my future
- I enjoy my friends a lot

- It's important for me to keep busy
- I hardly ever expect things to go my way
- I don't get upset too easily
- I rarely count on good things happening to me
- Overall, I expect more good things to happen to me than bad

Responses were structured as either “Agree”, “Disagree”, or “I don’t know”. There were minimal respondents who answered “I don’t know”, so respondents who answered negatively about their feelings were given a score of one for each statement. A higher score was indicative of a more negative feelings.

Quality control

Following recruitment and survey completion, each household was re-visited by a Quality Control Team to check whether (i) the tent was actually visited by the data collector, (ii) the female homemaker was interviewed whenever possible or was replaced by another member of the household, (iii) every working child (aged >8 to ≤ 18 years old) in the household was interviewed, and (iv) whether the month and year of birth for family members were reported according to legal papers.

Although our target sample size was 1,884 households, we sampled 1,907 eligible households of which four refused participation and one was vacant, leaving 1,902 in the final sample. The Quality Control Team detected and corrected inconsistencies in 8% (n=152) of the households, mainly relating to the year of birth of household members.

References

1. Habib RR, Mikati D, Hojeij S, El Asmar K, Chaaya M, Zurayk R. Associations between poor living conditions and multi-morbidity among Syrian migrant agricultural workers in Lebanon. *Eur J Public Health* 2016; **26**(6): 1039-44.
2. Habib RR, Yassin N, Ghanawi J, Haddad P, Mahfoud Z. Double jeopardy: assessing the association between internal displacement, housing quality and chronic illness in a low-income neighborhood. *Z Gesundh Wiss* 2011; **19**(2): 171-82.
3. Habib RR, Mahfoud Z, Fawaz M, Basma SH, Yeretzian JS. Housing quality and ill health in a disadvantaged urban community. *Public Health* 2009; **123**(2): 174-81.
4. Maxwell D, Caldwell R, Langworthy M. Measuring food insecurity: Can an indicator based on localized coping behaviors be used to compare across contexts? *Food Policy* 2008; **33**(6): 533-40.
5. World Food Programme. Consolidated Approach for Reporting Indicators of Food Security (CARI). [online] Available at: https://bit.ly/2JuH5rV [Date of access 17 July 2018]. 2014.
6. University of Miami. Life Orientation Test-Revised [online]. Available at: https://bit.ly/1n7DGUh [Date of access 17 July 2018]. 2007.

ADDITIONAL FILES

STROBE Checklist

STROBE Statement—Checklist of items that should be included in reports of ***cross-sectional studies***

	Item No	Recommendation	Location in manuscript
**Title and abstract**	1	(*a*) Indicate the study's design with a commonly used term in the title or the abstract	Abstract
		(*b*) Provide in the abstract an informative and balanced summary of what was done and what was found	Abstract
**Introduction**			
Background/rationale	2	Explain the scientific background and rationale for the investigation being reported	Page 4
Objectives	3	State specific objectives, including any prespecified hypotheses	Page 4
**Methods**			
Study design	4	Present key elements of study design early in the paper	Page 5, sub-heading "Design, setting, sampling"
Setting	5	Describe the setting, locations, and relevant dates, including periods of recruitment, exposure, follow-up, and data collection	Page 5, sub-heading "Design, setting, sampling"
Participants	6	(*a*) Give the eligibility criteria, and the sources and methods of selection of participants	Page 5, sub-heading "Design, setting, sampling"
Variables	7	Clearly define all outcomes, exposures, predictors, potential confounders, and effect modifiers. Give diagnostic criteria, if applicable	Page 5, sub-heading "Questionnaire and measures"<br>Supplementary Appendix
Data sources/ measurement	8*	For each variable of interest, give sources of data and details of methods of assessment (measurement). Describe comparability of assessment methods if there is more than one group	Page 5, sub-heading "Questionnaire and measures"<br>Supplementary Appendix
Bias	9	Describe any efforts to address potential sources of bias	Supplementary Appendix, sub-heading "Quality control"
Study size	10	Explain how the study size was arrived at	Page 5, sub-heading "Design, setting, sampling"

Quantitative variables	11	Explain how quantitative variables were handled in the analyses. If applicable, describe which groupings were chosen and why	Page 6, sub-heading "Statistical analysis"
Statistical methods	12	(*a*) Describe all statistical methods, including those used to control for confounding	Page 6, sub-heading "Statistical analysis"
		(*b*) Describe any methods used to examine subgroups and interactions	Page 6, sub-heading "Statistical analysis"
		(*c*) Explain how missing data were addressed	Page 6, sub-heading "Statistical analysis"
		(*d*) If applicable, describe analytical methods taking account of sampling strategy	Not applicable
		(*e*) Describe any sensitivity analyses	Not applicable
**Results**			
Participants	13*	(a) Report numbers of individuals at each stage of study—eg numbers potentially eligible, examined for eligibility, confirmed eligible, included in the study, completing follow-up, and analysed	Page 6<br>Supplementary Appendix
		(b) Give reasons for non-participation at each stage	Not applicable
		(c) Consider use of a flow diagram	Not completed
Descriptive data	14*	(a) Give characteristics of study participants (eg demographic, clinical, social) and information on exposures and potential confounders	Page 6
		(b) Indicate number of participants with missing data for each variable of interest	Tables 1-3
Outcome data	15*	Report numbers of outcome events or summary measures	Pages 6-7
Main results	16	(*a*) Give unadjusted estimates and, if applicable, confounder-adjusted estimates and their precision (eg, 95% confidence interval). Make clear which confounders were adjusted for and why they were included	Pages 6-7
		(*b*) Report category boundaries when continuous variables were categorized	Table 1, Table 3
		(*c*) If relevant, consider translating estimates of relative risk into absolute risk for a meaningful time period	Not applicable
Other analyses	17	Report other analyses done—eg analyses of subgroups and interactions, and sensitivity analyses	Not applicable
**Discussion**			
Key results	18	Summarise key results with reference to study objectives	

Limitations	19	Discuss limitations of the study, taking into account sources of potential bias or imprecision. Discuss both direction and magnitude of any potential bias	Page 14
Interpretation	20	Give a cautious overall interpretation of results considering objectives, limitations, multiplicity of analyses, results from similar studies, and other relevant evidence	Page 13-14
Generalisability	21	Discuss the generalisability (external validity) of the study results	Page 15
**Other information**			
Funding	22	Give the source of funding and the role of the funders for the present study and, if applicable, for the original study on which the present article is based	Abstract<br>Page 15

*Give information separately for exposed and unexposed groups.
